# Letter to the Editor Regarding “The microbiome of host saliva, gastric fluid, and gastric mucosa as accurate diagnostic tools for gastric cancer detection”

**DOI:** 10.1186/s12967-026-08514-2

**Published:** 2026-06-25

**Authors:** Yiming Cui, Yiheng Liu, Feifei Dou, Jingwei Mao

**Affiliations:** 1 First Affiliated Hospital of Dalian Medical University, Dalian City, Liaoning Province 116011 China; 2 The Second Affiliated Hospital of Dalian Medical University, Dalian City, Liaoning Province 116023 China

Dear Editor,

We read with great interest the recent article by Changzhen Lei et al., titled “The microbiome of host saliva, gastric fluid, and gastric mucosa as accurate diagnostic tools for gastric cancer detection” published in the ***Journal of Translational Medicine*** [1]. The authors conducted a prospective case-control study to systematically analyze microbiome differences in three ecological niches—saliva, gastric fluid, and gastric mucosa—between gastric cancer (GC) patients and healthy controls. They successfully employed machine learning algorithms to construct high-precision diagnostic models for GC. This study innovatively integrates information from the “oral-gastric” microbial axis, offering a promising new strategy for non-invasive early GC screening. Its rigorous design and excellent model performance are highly impressive.

The outstanding strength of this work lies in its integrated analysis of multi-niche microbiomes. Compared to previous studies often focusing on a single site (e.g., feces or gastric mucosa), this work simultaneously collected and compared microbial samples from the oral cavity (saliva) and stomach (fluid, mucosa). It revealed 13 differential bacterial genera shared by GC patients across these sites, with 9 showing concurrent alteration trends. This provides direct evidence for the synergistic role of the “oral-gastric” microbial axis in gastric carcinogenesis. Furthermore, the study employed a strategy combining Random Forest (RF) and Support Vector Machine (SVM) algorithms to screen biomarkers. The constructed diagnostic models demonstrated excellent performance in the training set (AUCs: 0.96, 0.87, 0.96) and showed robust validation in multiple external datasets, confirming their potential for clinical translation.

Although the study is well-designed and its conclusions are compelling, several important questions warrant further exploration to advance the field.

**First**,** it is about the impact of diet and population heterogeneity on model generalizability.** All participants in the study were of Han ethnicity from East China with a relatively homogeneous dietary pattern. Diet is a key environmental factor shaping the microbiome [2]. From our perspective, validation in diverse ethnic cohorts is needed. We suggest that future validation studies should proactively include populations with diverse dietary habits (e.g., high-salt, low-fiber, rich in fermented foods) and analyze how specific dietary components modulate the identified microbial diagnostic signatures. This would not only assess the model’s generalizability but also reveal the modulating role of diet-microbe interactions in GC risk.

**Second**,** from association to mechanism**,** the functional elucidation of diagnostic signatures is important.** This study, based on 16S rRNA data, predicted functional changes via PICRUSt2, but the authors rightly noted these are speculative hypotheses. How do the key identified genera (e.g., Prevotella, Streptococcusin saliva) concretely participate in gastric mucosal carcinogenesis [3]? We recommend subsequent multi-omics analyses on the stored samples, such as metagenomics and metabolomics. This would allow direct identification of microbial functional gene clusters (e.g., for toxin synthesis, mucin degradation, carcinogenic metabolite production) and associated host metabolites related to GC, thereby providing a more precise explanation of their carcinogenic or tumor-promoting mechanisms at the strain and functional pathway levels.

**Third**,** integration with existing biomarkers and cost-effectiveness analysis is one of the significant considerations forward clinical translation.** While the microbial diagnostic model performs excellently, what are the cost and accessibility implications of its use as a standalone screening tool? This is a crucial prospective direction. We propose designing a prospective cohort study to evaluate the diagnostic gain, cost-effectiveness, and patient acceptability of a “microbial signature panel **+** traditional serum tumor markers” combination in real-world early GC screening scenarios [4]. Additionally, exploring the performance of a simplified model based on a single, more accessible sample type (e.g., saliva only) would significantly enhance its feasibility for large-scale population screening.

**Linking microbial signatures to tumor biology is an innovative perspective.** We are curious whether the established microbial signatures strongly associated with GC (particularly gastric mucosa-related ones) correlate with specific clinicopathological features (e.g., Lauren classification, lymphovascular invasion, propensity for peritoneal metastasis) or patient prognosis [5]. Future research could further analyze the correlation between these microbial markers and features of the tumor immune microenvironment (e.g., CD8 + T cell infiltration, PD-L1 expression), exploring their potential in predicting immunotherapy response. This would extend the diagnostic value into the realms of prognostic assessment and treatment guidance.

Last but not least, we sincerely thank the team of Lei et al. for their outstanding work and the platform which the ***Journal of Translational Medicine*** provides for communicating. We look forward to future studies building upon these findings to ultimately translate the fundamental discoveries of the “oral-gastric” microbial axis into effective clinical tools that can improve outcomes for GC patients.

Sincerely,

All Authors

## Data Availability

Not applicable.

## Declarations

### Work statement

The work should be attributed to First Affiliated Hospital of Dalian Medical University, Dalian 116011, Dalian City, Liaoning province, China.

## Abbreviations

GC: Gastric Cancer
RF: Random Forest
SVM: Support Vector Machine
